# Autocrine activity of BDNF induced by the STAT3 signaling pathway causes prolonged TrkB activation and promotes human non-small-cell lung cancer proliferation

**DOI:** 10.1038/srep30404

**Published:** 2016-07-26

**Authors:** Bo Chen, Yan Liang, Zheng He, Yunhe An, Weihong Zhao, Jianqing Wu

**Affiliations:** 1 Department of Geriatrics, The First Affiliated Hospital of Nanjing Medical University, 300 Guangzhou Road, Nanjing 210029, China; 2 Department of Biotechnology, Beijing Centre for Physical and Chemical Analysis, 7 Fengxian Road, Beijing 10089, China

## Abstract

Brain-derived neurotrophic factor (BDNF) is a member of the neurotrophin superfamily, which has been implicated in the pathophysiology of the nervous system. Recently, several studies have suggested that BDNF and/or its receptor, tropomyosin related kinase B (TrkB), are involved in tumor growth and metastasis in several cancers, including prostate cancer, neuroblastoma, pancreatic ductal carcinoma, hepatocellular carcinoma, and lung cancer. Despite the increasing emphasis on BDNF/TrkB signaling in human tumors, how it participates in primary tumors has not yet been determined. Additionally, little is known about the molecular mechanisms that elicit signaling downstream of TrkB in the progression of non-small-cell lung cancer (NSCLC). In this study, we report the significant expression of BDNF in NSCLC samples and show that BDNF stimulation increases the synthesis of BDNF itself through activation of STAT3 in lung cancer cells. The release of BDNF can in turn activate TrkB signaling. The activation of both TrkB and STAT3 contribute to downstream signaling and promote human non-small-cell lung cancer proliferation.

Lung cancer continues to be the leading cause of cancer deaths worldwide. It can be divided into two major forms: non-small-cell lung cancer (NSCLC) and small cell lung cancer, which account for 80% and 20% of all lung carcinomas, respectively. The incidence of non-small-cell lung cancer (NSCLC) continues to rise, and its insensitivity to cytotoxic agents makes it critical to identify molecules that drive lung cancer growth, survival, and metastasis.

Brain-derived neurotrophic factor (BDNF) is a member of the neurotrophin superfamily, which has been implicated in the pathophysiology of the nervous system and is important for several neurological and psychological conditions^1^,^2^,^3^. Recently, several studies have shown that BDNF and/or its receptor, tropo-myosin-related kinase B (TrkB), are involved in cancer growth and metastasis in several cancers, including neuroblastoma^4^, pancreatic ductal carcinoma^5^, prostate cancer^6^, hepatocellular carcinoma^7^, and lung cancer^8^. However, a detailed understanding of the molecular mechanisms that elicit signaling downstream of TrkB in the progression of NSCLC is lacking.

Members of the signal transducer and activator of transcription (STAT) family of transcription factors are potential targets for the treatment and prevention of cancers, including non-small-cell lung cancer^9^,^10^,^11^,^12^. Signal transducer and activator of transcription 3 (STAT3) has long been shown to regulate gene transcription in response to cytokines and growth factors through JAK1^12^ or src-kinase^13^.

Studies have established STAT3 as a downstream mediator of Trk signaling and functions in PC12 cells and in the major pelvic ganglia (MPG) of rats^14^,^15^,^16^. However, it is not known whether STAT3 is also a mediator of BDNF/TrkB signaling in lung cancers. In this study, we report that BDNF stimulation increases the activation of STAT3, which in turn promotes the synthesis of BDNF in A549 and H1299 cells. We also show that the release of BDNF can in turn activate prolonged TrkB signaling.

## Results

### TrkB is constitutively activated in human lung cancers

**Figure 1:**
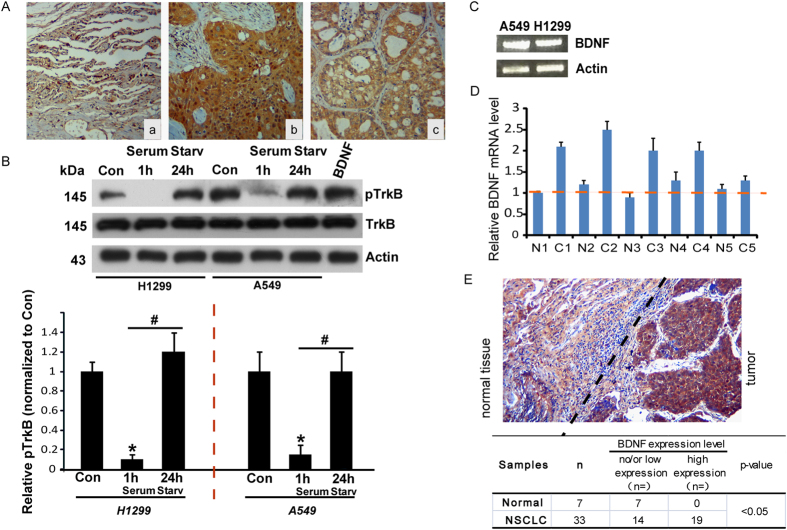
Constitutive activation of TrkB in human lung cancers. (**A**) Protein levels of TrkB were assessed by immunohistochemistry (100X; Brown as positive; a, normal tissue; b and c, cancer samples). (**B**) Levels of phosphorylated TrkB under indicated conditions were analyzed in A549 and H1299 cells. Cell lysates were immunoblotted with anti-phosphoTrkB and anti-Total TrkB antibodies. β-actin was used as a loading control. Cell lysate pretreated with BDNF was used as a positive control. The relative levels of pTrkB were quantified (down). Results are shown as the mean ± S.E. **n** = **3**; *p < 0.05, vs Control group; ^#^p < 0.05, vs serum starvation for 1 h group; one-way ANOVA. (**C**) Expression of BDNF was analyzed in A549 and H1299 cells by RT-PCR. β-actin was used as a control. (**D**) BDNF mRNA levels from lung cancer tissues were analyzed using real-time PCR; N: Normal tissue; C: Cancer samples. (**E**) Protein levels of BDNF were assessed by immunohistochemistry (100X; Brown as positive; left, normal tissue; right, tumor tissue).

We tested the expression of TrkB in 33 NSCLC specimens by immunohistochemical assay. We observed that in 21/33 (64%) samples, the expression of TrkB was higher (with more than 60% positive cells) in tumor samples than in adjacent normal controls (~15% positive cells) (Fig. 1A). To characterize TrkB expression and activation status *in vitro*, we examined A549 and H1299 cell lysates under different conditions by Western blot. Lysate from A549 cells pretreated with BDNF (25 ng/ml) for 1 h was used as a positive control. We observed the expression and activation of TrkB in both A549 and H1299 cells, as shown in Fig. 1B. The level of phosphorylated TrkB decreased upon serum starvation for 1 h but returned to its basal level upon serum starvation for 24 h in A549 and H1299 cells. The spontaneous recovery of phosphorylated TrkB under serum-deprived conditions suggested the presence of an autocrine mechanism. To confirm this possibility, we investigated the expression of BDNF, the ligand of TrkB receptors, in A549 and H1299 cells by RT-PCR (Fig. 1C). The results indicated endogenous expression of BDNF. A BDNF ELISA further confirmed detectable levels of BDNF secretion in the media of A549 and H1299 cells (26.6 ng/ml and 63.2 ng/ml, respectively). To confirm the *in vitro* results, we measured BDNF levels in a panel of NSCLC samples containing normal tissue by real-time PCR. As shown in Fig. 1D, we detected significantly increased levels of BDNF transcripts in most cancer samples (4 of 5) compared with normal tissues. We also tested the expression of BDNF in 33 NSCLC specimens by immunohistochemical assay and found that in 19/33 (57%) samples, the expression of BDNF was higher in the tumor samples than in the adjacent normal controls (Fig. 1E). What is more, the co-expression of BDNF (namely BDNF+/TrkB+) was found in 54.5% (18 out of 33) TrkB positive samples; the percentage of BDNF-/TrkB+ was 6.0%; the percentage of BDNF+/TrkB- was 3.0%; the percentage of BDNF-/TrkB- was 33.3% respectively. These results strongly suggest that the activation of TrkB is common in NSCLC and is induced by the secretory factor BDNF.

### BDNF is a major regulatory factor of STAT3 activation in lung cancer cells

Signal transducer and activator of transcription 3 (STAT3) has long been shown to regulate gene transcription and play a role in the progression of NSCLC^9^,^10^,^11^,^12^. A previous study reported STAT3 as the downstream signaling target of BDNF/TrkB^14^,^15^,^16^.

**Figure 2:**
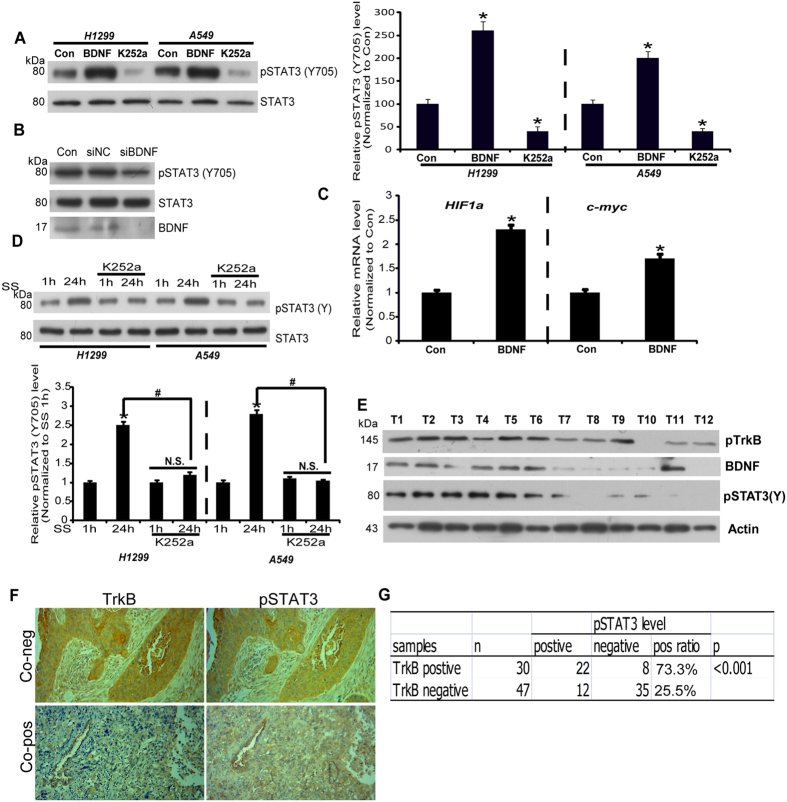
BDNF regulates STAT3 activation. (**A**) Phosphorylation of STAT3 (Y705) under indicated conditions was analyzed in A549 and H1299 cells. Cell lysates were immunoblotted with anti-phospho STAT3 (Y705) and anti-Total STAT3 antibodies. The relative levels of pSTAT3 (Y705) were quantified (right). Results are shown as the mean ± S.E. n = 3; *p < 0.05, one-way ANOVA. (**B**) Phosphorylation of STAT3 (Y705) under indicated conditions was analyzed in A549 cells. Cell lysates were immunoblotted with anti-phospho STAT3 (Y705) and anti-Total STAT3 antibodies. The knockdown efficiency of BDNF siRNA was detected by WB of BDNF. (**C**) The relative expression levels of HIF1a and c-Myc upon BDNF treatment were detected by Real-time PCR. Results are shown as the mean ± S.E. n = 3; *p < 0.05, vs Control group, Student’s t-test. (**D**) Recovery of phosphorylation levels of STAT3 (Y705) under indicated conditions was analyzed in A549 cells. Cell lysates were immunoblotted with anti-phospho STAT3 (Y705). The relative levels of pSTAT3 (Y705) were quantified (down). Results are shown as the mean ± S.E. n = 3; *p < 0.05, vs serum starvation for 1 h group; ^#^p < 0.05, vs K252a untreated group; one-way ANOVA. (**E**) The relative levels of pSTAT3 (Y705) and pTrkB were detected by immunoblot in lung cancer tissues. β-actin was used as a loading control. (**F**,**G**) Expression of TrkB and pSTAT3 (Y705) was detected in NSCLC samples by immunohistochemistry (100X; Brown as positive).

To study whether BDNF/TrkB signaling regulates the activation of STAT3 in lung cancer cells, we examined the level of phosphorylated STAT3 in cells with or without the Trk inhibitor K252a (100 nM). The results indicated that blocking TrkB activity decreased STAT3 phosphorylation at tyrosine705 (Y705), which enhanced the transcriptional activity of STAT3. Treatment with BDNF (50 ng/ml) resulted in further activation of STAT3 in both A549 and H1299 cells (Fig. 2A). Furthermore, as shown in Fig. 2B, BDNF knockdown by siRNA resulted in significantly reduced STAT3 phosphorylation at the tyrosine residue (~0.5 fold; n = 3, p < 0.05, student’s *t*-test). To confirm the change in the transcriptional activity of STAT3, we investigated the transcript levels of c-Myc and HIF1a, both of which are well-known target genes of STAT3^17^,^18^, by qPCR and observed that BDNF increased the mRNA levels of c-Myc and HIF1a in A549 cells (Fig. 2C). This result indicated that BDNF was a regulator of STAT3 in lung cancers. To confirm this possibility, A549 and H1299 cells were serum-deprived and treated with K252a for 24 h, followed by measurements of STAT3 phosphorylation levels under the aforementioned conditions. The results showed that blocking TrkB activity with K252a reduced the spontaneous recovery of STAT3 activation in A549 and H1299 cells under serum-deprived conditions (Fig. 2D). Furthermore, we examined the levels of phosphorylated STAT3 and TrkB in 8 NSCLC samples by Western blot. As shown in Fig. 2E, we observed concurrent upregulation of STAT3 activity in the samples that exhibited TrkB activation. To further confirm the result got by Western blotting, we performed immunohistochemical assay in 77 NSCLC specimens and detected the relationship between the STAT3 activity and TrkB expression. As shown in Fig. 2F,G, the p-STAT3 positive rate in TrkB-positive tissue was 73.3%, much higher than the rate in TrkB-negative tissue. The above-mentioned findings strongly suggest that BDNF functions partially as a secretory factor that induces STAT3 activation in lung cancer.

### STAT3 regulates BDNF expression in lung cancer cells

**Figure 3:**
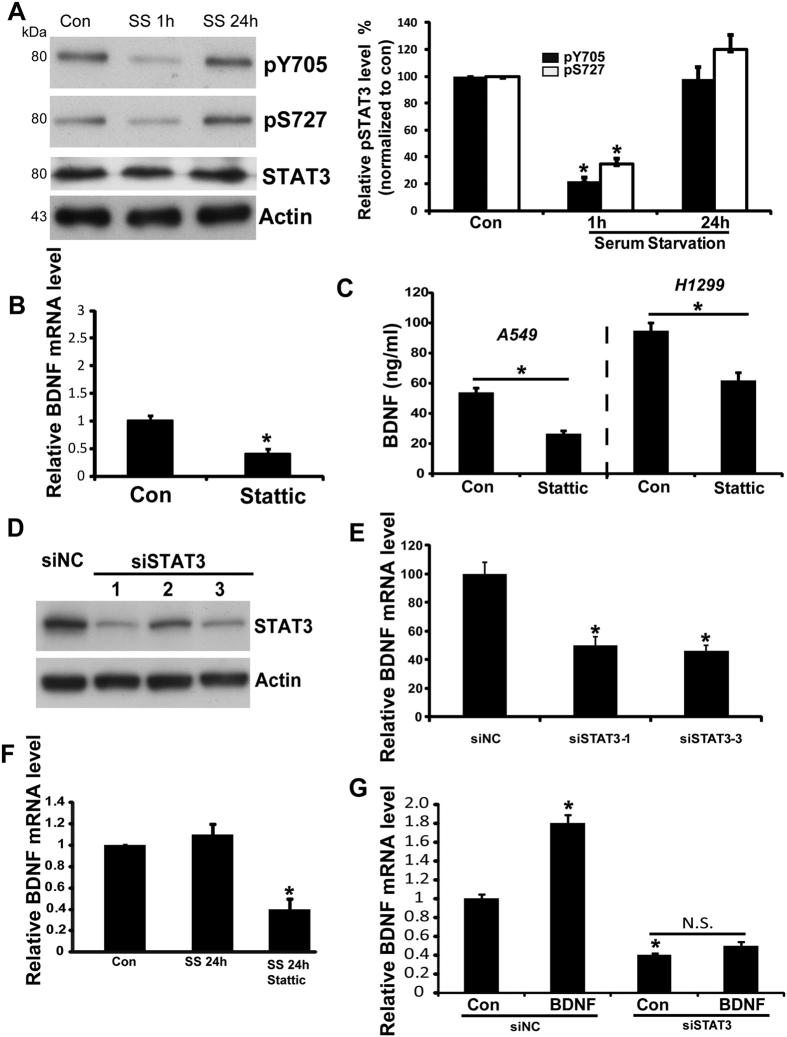
STAT3 is the major mediator of BDNF expression. (**A**) Phosphorylation of STAT3 (S727) (Y705) under indicated conditions was analyzed in A549 cells; SS, serum starvation. Cell lysates were immunoblotted with anti-phospho STAT3 (S727) (Y705) and anti-Total STAT3 antibodies. β-actin was used as a loading control. The relative levels of pSTAT3 (S727) (Y705) were quantified (right). Results are shown as the mean ± S.E. **n** = **3**; *p < 0.05, vs Control group, Student’s t-test. (**B**) BDNF mRNA levels were analyzed using real-time PCR in A549 cells treated with or without ST. Results are shown as the mean ± S.E. n = 3; *p < 0.05, vs Control group, Student’s t-test. (**C**) BDNF secretion was analyzed using ELISA in A549 and H1299 cells treated with or without Stattic. Results are shown as the mean ± S.E. n = 3; *p < 0.05, vs Control group, Student’s t-test. (**D**) The knockdown efficiency of STAT3 siRNA in A549 cells is shown. (**E**) BDNF mRNA level was analyzed using real-time PCR in A549 cells transfected with STAT3 siRNA or siNC. Results are shown as the mean ± S.E. n = 3; *p < 0.05, vs siNC group, Student’s t-test. (**F**) BDNF mRNA levels were analyzed using real-time PCR in A549 cells under indicated conditions. Results are shown as the mean ± S.E. n = 3; *p < 0.05, vs the SS 24 h group, Student’s t-test. (**G**) BDNF mRNA levels were analyzed using real-time PCR in A549 cells under indicated conditions. Results are shown as the mean ± S.E. n = 3; *p < 0.05, vs siNC–con group; Student’s t-test.

To investigate whether STAT3 can, in turn, serve as a mediator of BDNF expression in lung cancer cells, we analyzed the level of phosphorylated STAT3 under serum-deprived conditions by Western blot. We observed increased levels of STAT3 phosphorylation at tyrosine705 (Y705) in A549 cells that were serum starved for 24 h compared with the control group. We also detected similar results for Ser phosphorylation (S727) of STAT3 (Fig. 3A). The spontaneous recovery of STAT3 activation in A549 cells under serum-deprived conditions was in line with the time course of TrkB activation. Furthermore, BDNF mRNA levels were reduced upon pre-treatment of the cells with the STAT3 specific inhibitor, Stattic (20 μM) (Fig. 3B). The BDNF ELISA further confirmed that BDNF secretion was reduced in the media of A549 and H1299 cells upon Stattic treatment (Fig. 3C). To eliminate the potential non-specific effects of Stattic, we knocked down STAT3 expression by siRNA. As shown in Fig. 3D, the #1 and #3 siRNA could reduce the protein level of STAT3 to 32% and 35% respectively (n = 3, p < 0.05, one-way ANOVA) And the observed results of Q-PCR were consistent with Stattic treatment (Fig. 3D,E). Additionally, we observed that inhibition of STAT3 activity by Stattic under serum starvation blocked the recovery of BDNF in A549 and H1299 cells (Fig. 3F). Furthermore, we found that BDNF stimulation increases the transcription of BDNF itself, whereas siSTAT3 blocks BDNF transcription (Fig. 3G). Thus, the above-mentioned results indicate that STAT3 can serve as a mediator of BDNF expression in lung cancer.

### Activation of STAT3 and TrkB is involved in Akt activation and promotes human non-small-cell lung cancer cell proliferation

**Figure 4:**
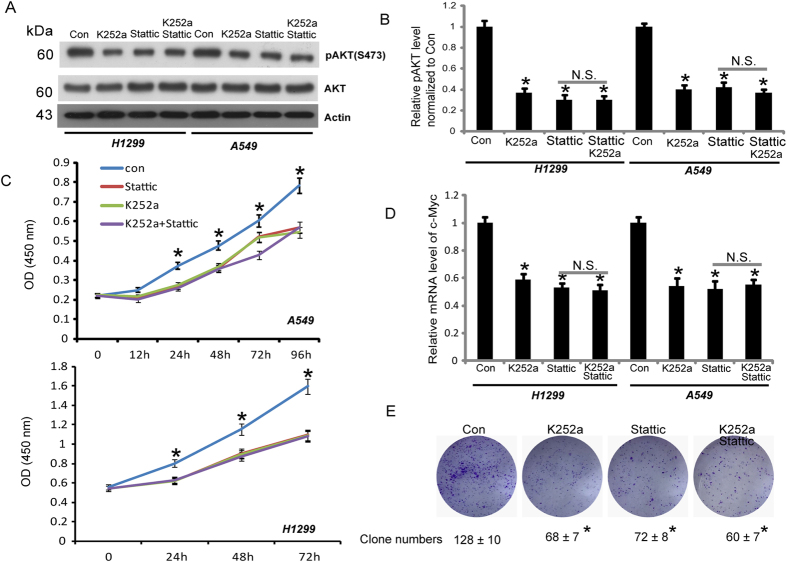
STAT3 and TrkB activation are involved in the activation of AKT and promote human non-small-cell lung cancer proliferation. (**A**) Phosphorylation of (S473) under indicated conditions was analyzed in A549 and H1299 cells. Cell lysates were immunoblotted with anti-phosphor-AKT (S473) and anti-Total AKT antibodies. β-actin was used as a loading control. (**B**) Quantification of phospho-AKT (S473) levels shown in A. Data are shown as the mean ± S.E. n = 3, *p < 0.05. One-way ANOVA. (**C**) CCK-8 assay for A549 and H1299 cells treated with different inhibitors. Data are shown as the mean ± S.E. n = 3, *p < 0.05. One-way ANOVA. (**D**) Effects of K252a and Stattic on c-Myc transcription by real-time PCR and quantitation of c-Myc mRNA levels are shown. Data are shown as the mean ± S.E. n = 3, *p < 0.05. One-way ANOVA. (**E**) The colony formation assay was further performed in A549 cells. (Data are shown as the mean ± S.E. n = 3, *p < 0.05. One-way ANOVA.)

Activation of Akt is a key event during the survival and proliferation of cells exposed to apoptotic stimuli, such as serum deprivation, and frequent hyperactivation of Akt signaling is a well-established phenomenon in several human cancers^19^,^20^,^21^,^22^,^23^,^24^,^25^. To examine whether STAT3 and TrkB activation are associated with the activation of Akt, we analyzed Akt phosphorylation at Serine473 (S473) by inhibiting endogenous TrkB and STAT3 activity in A549 and H1299 cells using K252a and Stattic, respectively. As shown in Fig. 4A,B, Akt phosphorylation was decreased by Stattic or K252a treatment. These results indicated that both BDNF/TrkB and STAT3 activity were involved in the constitutive activation of Akt. However, the co-addition of K252a and Stattic did not further decrease Akt phosphorylation, indicating that STAT3 and TrkB activation shared the pathway involved in the regulation of Akt in A549 and H1299 cells. To investigate the potential role of STAT3 and TrkB activation in the biology of lung cancer, we analyzed the rates of A549 and H1299 cell proliferation upon treatment with Stattic or/and K252a by CCK8 assay. As shown in Fig. 4C, cell growth was inhibited upon treatment with Stattic (20 μM) or/and K252a (100 nM), with no significant difference between single or combination drug treatment. To further confirm the CCK8 results, we examined the expression of the proliferation-related gene c-Myc and observed that c-Myc expression was significantly reduced upon treatment of the cells with Stattic or/and K252a, with no further reduction upon combining these treatments (Fig. 4D). The colony formation assay was further performed to confirm the result in A549 cells. As shown in Fig. 4E, the colony-forming activity was inhibited upon treatment with Stattic (10 μM) or/and K252a (100 nM), with no significant difference between single or combination drug treatment. All the results indicated that activation of both STAT3 and TrkB was involved in the activation of Akt and the promotion of human non-small-cell lung cancer proliferation, with STAT3 and TrkB sharing the same regulation pathway.

## Discussion

Lung cancer remains the leading cause of cancer deaths worldwide, and the incidence of non-small-cell lung cancer (NSCLC), the main form of lung cancer, continues to rise. Due to its insensitivity to cytotoxic agents, the identification of molecules that drive lung cancer growth, survival, and metastasis is critical.

BDNF is a member of the nerve growth factor family and is important in the phenotypic behavior of neurons, as well as nervous system cancers^1^,^3^,^4^,^26^. BDNF mediates its biological effects in cells mainly through a tyrosine kinase receptor, TrkB. The expression and activity of TrkB has been reported in several types of carcinomas, although its mechanism of action remains to be elucidated^4^,^5^,^6^,^26^,^27^. The expression of TrkB and BDNF is associated with a poor prognosis in NSCLC patients. Although BDNF/TrkB signaling is involved in tumorigenicity and malignant progression to invasiveness in large cell neuroendocrine carcinoma (LCNEC) and may be a potential target in LCNEC^26^,^27^, how this signaling is activated is not well understood.

In this study, our results suggest that activation of STAT3 can drive BDNF expression and that BDNF stimulation can further increase its own synthesis through the activation of STAT3 in A549 cells. The release of BDNF can in turn activate downstream TrkB signaling. The autocrine regulation of BDNF in A549 and H1299 cells may contribute to its biological function. Our results provide several new insights into the regulation of BDNF expression, as well as its signaling mechanism.

First, we showed that the activation of TrkB in A549 cells was triggered by autocrine regulated BDNF, which was dependent on STAT3 activity. According to previously published reports and our data, TrkB is overexpressed and abnormally activated in lung cancer, and exogenous BDNF increases TrkB activation and affects its downstream function. However, the *in vivo* source of BDNF is unknown. By employing RT-PCR, immunohistochemistry and ELISA, we determined that endogenously expressed BDNF is involved in the regulation of TrkB activity in A549 and H1299 cells. Additionally, we found that BDNF expression was under the control of STAT3 and was decreased upon the inhibition of STAT3 activity by Stattic in A549 and H1299 cells, thus establishing STAT3 as a critical regulator of BDNF in lung cancer cells.

Second, we showed that BDNF/TrkB signaling can further up-regulate the activation of STAT3, which can in turn increase BDNF expression and TrkB activation. In other words, STAT3 can regulate the expression of BDNF, which can in turn control STAT3 activity in a feedback loop. Thus, BDNF-regulated STAT3 activation can further strengthen BDNF expression, which may be the reason for enhanced STAT3 and TrkB activation in lung cancer.

Third, we demonstrated that STAT3 and TrkB activity is involved in the activation of PI3K-Akt signaling. Our results showed that inhibition of STAT3 and TrkB activity using Stattic and K252a, respectively, reduces Akt activity in A549 and H1299 cells. However, co-treatment with the two inhibitors does not further decrease Akt activation. These results suggest that STAT3 and TrkB activation is involved in the regulation of Akt activity in lung cancer cells.

In summary, we report that STAT3 activity triggers BDNF expression and TrkB activation, which in turn enhances the activity of STAT3 and further strengthens BDNF expression and TrkB activation, as well as downstream signaling pathways, such as PI3K-Akt. STAT3 and TrkB activity may contribute to non-small-cell lung cancer cell proliferation.

## Materials and Methods

### Tissue microarray

We purchased a lung cancer tissue microarray from Shanghai Zhuoli Biotechnology Co., Ltd (Zhuoli Biotechnology Co, Shanghai, China), which contained 37 informative specimens (including 4 noncancerous lung tissues as controls). Analysis of these specimens is described in each experimental method.

### Immunohistochemistry

Representative formalin-fixed and paraffin embedded tissue sections (6 mm thickness) were used for immunohistochemistry with specific antibodies to BDNF (Santa Cruz Biotechnology,,CA) or TrkB (Millipore, Temecula, CA). Slides were deparaffinized with xylene, rehydrated in descending concentrations of ethanol and boiled in citrate buffer for 10 min. Endogenous peroxidase activity was suppressed with 3% H_2_O_2_ for 10 min. The slides were serum-blocked (goat serum) and incubated with BDNF antibody (1:100) or TrkB antibody (1:100) for 1 h at room temperature and then stained with an ElivisionTM plus Polyer HRP (Mouse/Rabbit) (Fuzhou Maixin Biotech. Co., Ltd) IHC Kit according to the manufacturer’s instructions. For the negative controls, all conditions remained the same, except that the first antibody was eliminated.

### Cell culture and treatments

Human pulmonary epithelial cell lines A549 and H1299 were maintained in Dulbecco’s modified Eagle’s medium (Invitrogen, Carlsbad, CA, USA) supplemented with 10% fetal bovine serum (Invitrogen) and 5 mM glutamine and cultured at 37 °C in a humidified atmosphere of 5% CO_2_. The cells were seeded in six-well plates at 80% confluence.

### Western blot

Protein levels were measured by Western blot. Briefly, equal amounts of protein (10 μg per lane) were separated by SDS-PAGE and transferred to PVDF membranes (Bio-Rad Inc.). The membranes were incubated in blocking buffer (0.2 mM Tris, 137 mM NaCl, 5% non-fat milk, and 0.1% Tween-20) for an hour and then probed at 4 °C overnight with KL antibody. The membranes were rinsed with washing buffer (0.1% Tween 20, 0.2 mM Tris, and 137 mM NaCl) and incubated with HRP-conjugated secondary antibody (1:5000) for 1 hour at room temperature, followed by chemiluminescent detection. For densitometric analyses, immunoreactive bands were scanned and quantitated using NIH ImageJ (Scion,Frederick,MD). All experiments were carried out at least in triplicate. And the representative blots were shown in the figures.

### Quantitative RT-PCR

Transcription of BDNF was measured by quantitative RT-PCR. Cells were washed with PBS, followed by RNA extraction. Total RNA was extracted with TRIzol-A+ RNA isolation reagent (TIANGEN) according to the manufacturer’s instructions. Reverse transcription was performed with 1 μg of total RNA and a RevertAid First Strand cDNA Synthesis Kit (Fermentas). The primer sequences were as follows: BDNF forward primer: 5′-GAC ATC ATT GGC TGA CAC TTT-3′ and reverse primer: 5′-TAC TGA GCA TCA CCC TGG AC-3′; c-Myc forward primer: 5′-CCA CCT CCA GCT TGT ACC TG -3′ and reverse primer: 5′-GAG CAG AGA ATC CGA GGA CG-3′; and HIF1a forward primer: 5′-TTT TGG CAG CAA CGA CAC AG-3′ and reverse primer: 5′- TGA TTG AGT GCA GGG TCA GC-3′. Primers specific for β-actin were used as a control (forward: 5′-TGA CGT GGA CAT CCG CAA AG-3′ and reverse: 5′-CTG GAA GGT GGA CAG CGA GGT-3′). Quantitative RT-PCR was performed in a cycler (MyiQ2, Bio-Rad) using SYBR green (Roche). The threshold cycle for each sample was chosen from the linear range and converted to a starting quantity by interpolation from a standard curve run on the same plate for each set of primers. BDNF mRNA levels were normalized for each well to β-actin mRNA levels using the 2-ΔΔCT method. Each experiment was repeated three times.

### CCK8 assay

A549 cell proliferation, under the above-mentioned conditions, was measured using a CCK-8 assay, which is based on the conversion of a water-soluble tetrazolium salt, 2-(2-methoxy-4-nitrophenyl)-3-(4-nitrophenyl)-5-(2,4-disulfophenyl)-2H-tetrazolium, monosodium salt (WST-8), to a water-soluble formazan dye upon reduction by dehydrogenases in the presence of an electron carrier. A549 cells (2 × 10^4^ cells/ml) were grown in 96-well plates for 48 h and treated with the indicated inhibitors. At different time points, the cells were washed, and the extent of cell growth was assessed using the CCK-8 assay (Solarbio, China). CCK-8 solution (10 μl) was added to each well, followed by incubation for 2 h at 37 °C. A multiplate reader was used to measure absorbance at 450 nm. Cell viability was expressed as a percentage of the control (untreated) cells.

### Enzyme-linked immunosorbent assay (ELISA)

A549 or H1299 cells (4.5 × 10^5^) were seeded into 6-well plates. Six hours before drug addition, the cells were placed in DMEM (without FBS) and then treated with or without Stattic for 12 h. BDNF protein concentrations in the respective media samples were determined using the BDNF Human ELISA (Enzyme-Linked Immunosorbent Assay) kit (Abcam), with recombinant BDNF serving as the standard. Standards and samples were analyzed in duplicate, and each group contained 3 independent samples.

### Colony formation assay

A549 cells were trypsinized, counted, plated in a 6-well plate at a density of 100 cells/well and incubated at 37 °C for 14 days. The culture medium was changed every 2 days. The cell colonies were fixed with 4% paraformaldehyde for 10 min and stained with crystal violet for 20 min. Each cell colony was counted under a light microscope and photographed sing a digital camera. All samples had three repetitions.

### Statistical analysis

Statistical significance was assessed using Student’s *t-*test or analysis of variance (ANOVA). Data are presented as the mean ± SEM, and p < 0.05 was considered significant. All experiments were performed in triplicate.

## Additional Information

**How to cite this article**: Chen, B. *et al*. Autocrine activity of BDNF induced by the STAT3 signaling pathway causes prolonged TrkB activation and promotes human non-small-cell lung cancer proliferation. *Sci. Rep.* **6**, 30404; doi: 10.1038/srep30404 (2016).
